# Reasons for Endorsing the Hand Mallet

**Published:** 1910-05-15

**Authors:** E. K. Wedelstaedt

**Affiliations:** St. Paul, Minn.


### REASONS FOR ENDORSING THE HAND MALLET.

BY DR. E. K. WEDELSTAEDT, ST. PAUL, MINN.

It may be well on the start to remember that there are other means of condensing gold besides using a plugger and an assistant manipulating the mallet. Men who use mechanical mallets often become expert. And perhaps these men might not be able to condense gold as thoroughly by other means as by using the mechanical mallet. From this you can readily judge that it is my intention to view this subject from an unbiased and unprejudiced standpoint, and in as fairminded a way as possible. But, bear this in mind, facts are cold things and frequently they are not to our liking.

Whatever may be said, remember that we are dealing with some things which you may prove. It is not a good plan to condemn the ideas of other men with too great severity, until we prove that they are incorrect. Then, and not until then, are we in a position to know and know why we know.

I endorse the use of the hand mallet; 1st. Because I am used to that method of operating. 2nd. Because it is the most definite of all methods for condensing gold. 3rd. Because it is the most expeditious of all methods. 4th. Because more gold can be condensed into a cavity by its use than where other means of condensing are used. 5th. Because the hand mallet is at all times under perfect control of the assistant. 6th. Because it is the easiest of all methods for the patient. 7th. Because I believe it to be far superior to any of the mechanical mallets.

Here are seven reasons which are worthy of our consideration:

1st.—We become used to some method of operating. Habits are not easy things to break one's self of. Therefore, the hand mallet is preferred because I am used to that means of operating. Every man in this audience has his individual preference for the means which he uses for condensing gold. Dr. Maercklein endorses and uses the electric mallet. He feels that there is nothing else which he could use that would be so satisfactory. On the other hand, Dr. Southwell feels that when it comes to condensing gold there is nothing which he could use that would be so satisfactory, both to himself and his patients, as the Bonwill Mechanical Mallet. There are any number of other gentlemen who will smile at all such statements, because they believe some other sort of mechanical mallet is the best there is. We are creatures of habit, and right or wrong, we too often instinctively follow or stick to some things which it would be well for us all to rid ourselves of.

2nd.—The mallet in the hands of an assistant gives results which are as definite as it is possible for a person to obtain. To obtain certain results we wish a two pound blow delivered on the plugger, while condensing the gold in one portion of the cavity, while in another portion of the cavity, a ten pound blow may be necessary. With the trained assistant this is possible.

For a number of years it has been my teaching to use a given number of blows on pieces of gold foil of definite sizes. Thus a pellet made from one thirty-second of a sheet of gold, should receive not less than forty blows from the hand mallet. A pellet made from a sixteenth of a sheet, should receive not less than eighty blows, etc. Where this plan is followed the result is something definite. The blows are counted as they are delivered and thus the operator knows exactly what he is doing.

If, as we have been led to believe, the dentine is resilient and the best results are obtained by springing the dentine —I say if we believe this—then there is no other means, insofar as this relates to mallet force, for us to use in condensing gold, but a mallet in the hand of an assistant. Most certainly, if a man does not believe that the dentine is resilient, he is not in a position to understand the meaning contained in this statement.

3rd.—It is the most expeditious of all methods, for the reason that an ordinary plugger can be stopped more easily and used more quickly than it is possible to use any of the mechanical mallets. It is also far easier to wedge the gold and "send it home" than by using any of the mechanical mallets. Much tighter joints can be made than by condensing the gold with any of the mechanical mallets.

4th.—Because more gold can be condensed into a cavity by its use than where other means of condensing are used.

This is the part of the essay that is difficult for me to bring to your attention. Altogether too many men are cut on the bias of early acquisition, and on this account they do not wish to be disturbed in what they feel is the security of their belief. Woe betide that man who disturbs our peace or what we may feel is our peace. Suppose that we take the steel cavity block, with a cavity 100 by 143 thousandths (cylinder) and make some experimental fillings, using for this purpose thirty-seconds, i. e., a sheet of number four gold foil which is cut into thirty-two pieces, and each piece is rolled into pellet form. Each piece is annealed prior to being placed in the cavity. What shall result? Let us see.

1st. Hand mallet, 32nds, 50 blows on each piece, 5mm. straight plugger. Weight of filling, 611 milligrams.

2nd. Same as above, but 1mm. 3 degrees round plugger. Weight of filling, 514 milligrams.

3rd. Automatic mallet, short blow, full force, 25 blows on each piece. Weight of filling, 332 milligrams.

4th. Same as above, 50 blows given each piece. Weight of filling, 406 milligrams.

5th. Bonwill Mechanical mallet, serrated plugger, 25 sec. malleting. Weight of filling, 460 milligrams.

6th. Same as above. smooth plugger used. Weight of filling, 460 milligrams.

7th. Electric mallet, 25 sec. malleting given each piece. Weight of filling, 279 milligrams.

8th. Same as above, but 35 sec. malleting given each piece. Weight of filling, 358 milligrams.

Let us consider what these results mean and what bearing they have on our operations.

In operating on the human teeth it should be the aim of every man to condense the gold as thoroughly as possible. In condensing the gold the dentine should be slightly sprung so that it will grasp the gold. The gold should be condensed and the cavity filled. Now let us proceed a step farther. I took a cavity in a steel block. The cavity was in the form of a cylinder. Its size was 100 by 143 thousandths of an inch. Every filling was made in the cavity named. Two men, who for years had used mechanical mallets made fillings for me. I also made fillings. Each operator used the same sized pellets, i. e., 32nds of a sheet of No. 4 gold foil for his operation. Notwithstanding this, there was a variation of from 15 to 75 per cent in the amount of gold the men used in making their operations. Now gentlemen, there must be something wrong with either the men, the methods which they used or else with the condensing power of the mechanical mallets.

Before I go on, allow me to say that I did not come here seeking a controversy. I came here seeking the truth. It is very true I have brought you the results of the weighings of these different fillings, but I have done this purposely. Perhaps some of you gentlemen have made similar experiments, and I should like to compare results with you.

Let us return once more to the results and let them teach us the lesson which years ago, we should have learned so that by now we would "know it by heart" as the boys say.

There were two fillings condensed with the hand mallet. Weight of fillings, 1,136 milligrams.

There were two fillings condensed with the automatic mallet. Weight of fillings, 738 milligrams.

There mere two fillings condensed with the Bonwill Mechanical mallet. Weight of fillings, 920 milligrams.

There were two fillings condensed with the Electric mallet. Weight of fillings, 637 milligrams.

Here is a difference in the results of from 15 to 75 per cent. In other words 15 per cent more gold was condensed in the cavity by using a hand mallet than was condensed in it with the Bonwill Mechanical Mallet. From 33 to 35 per cent more gold was condensed in the cavity with the hand mallet than was condensed in it with the automatic mallet.

From 43 to 75 per cent more gold was condensed in the cavity with the hand mallet than was condensed in it with the electric mallet. (It is no longer any wonder to me, that those who assert that they are experts in the use of the electric mallet have for years and years continually talked about the "Stretching of gold under the stress of occlusion," a thing which I have never observed where gold was condensed with the hand mallet.)

I do not assert that these results are infallible. I merely tell you these results are what were obtained, that the results may be relied on and that they were obtained in good faith. Until they are proved incorrect they should be considered standard.

Take these fillings which are being condensed with the electric mallet, stop right in the middle of the condensing and observe what a plugger and hand mallet will do. Why, for over twenty years many men have known what results. I am neither biased nor prejudiced regarding the use of any mallet, but I am like the man from Missouri, I wish to be shown, provided you have something better than I am using.

Let us consider for just a moment how stress, in the form of extra blows or malleting, influenced the results as they were obtained. In experiment No. 3 each pellet received but twenty-five blows from the automatic mallet. Weight of filling, 332 milligrams.

In experiment No. 4 each piece received fifty blows, and the result was: Weight of filling, 406 milligrams.

Practically the same results were obtained in experiment Nos. 7 and 8. In the two last named experiments ten additional seconds of malleting were given each piece of gold and seventy-nine additional milligrams of gold were placed in the cavity.

Although I have made upwards of one hundred experimental fillings and doubtless I have as many more, which other men have made for me, the results are most unsatisfactory to me personally. What is necessary to do is this: To go into greatest detail by making similar experiments by using pluggers of various forms and sizes with different sized pellets. I have not the time just now, in which to do this. Your meeting is barely a week from the present writing and as it is, I am hardly able to find time in which to write out my ideas much less begin a new series of experiments which are so essential and necessary, provided, we wish all the data as they relate to this subject.

The men who made the experiments with the mechanical and electric mallets protested against using pellets of gold of a different sort. The result was amusing. The filling which was made with the mechanical mallet, no record being kept as to the time of malleting, weighed but 452 milligrams.

The filling which was made with the electric mallet weighed 386 milligrams.

In one case a lighter filling resulted and in the other a heavier filling was obtained than those made for the experiment, per se.

5th. Because the hand mallet is at all times under the perfect control of the assistant.

A well trained assistant has absolute control of the mallet which she uses. She takes greatest pride in her ability to do her best, and she makes it her business to be of worth to her employer. If, while malleting, she is inattentive and utterly unambitious—a clock studier—as some assistants are, she is a nuisance and better rid of than kept in an office.

6th. From conversation with patients I have been led into believing that the hand mallet suits, by far, the majority of patients much better than any other sort of malleting.

7th. Because the hand mallet is far superior to any of the mechanical mallets.

The mallet, in the hand of an educated and intelligent assistant, is the best possible means of obtaining just what is necessary for condensing gold, i. e., live blows. I know of nothing that is used in the form of mechanical mallets that delivers such blows. On the contrary every form of mechanical mallet, excepting the electric, delivers a dead blow, it is a thud, and a thud is never desired.

The electric mallet does not deliver blows but "pats." It does not weld the gold, it merely pats it down. If this statement is doubted it is a very simple matter to prove it. The results of the experiments to which I have called your attention also prove it.

In condensing gold the object is to weld it, provided that I understand the question. If such is the case, it might be well if those who are interested in this subject would watch the welding of steel and observe what manner of means are used to obtain the best results.

A plugger is much easier to handle than is any of the mechanical mallets. The operator's mind can be concentrated on what he is doing and his movements are not hampered. With a plugger he has much more freedom than where using mechanical mallets of any nature. It is a superior method for the reason that harder and denser fillings may at all times be made.

What we most need, is definite knowledge, not definite knowledge along one line, but definite knowledge along all lines. Take this matter of condensing gold for an example. How much is known about it? Well, not very much and it often seems to me, that few men care to know anything about it. Let me illustrate this: A short time ago while I was standing beside the chair of a prominent dentist, he was observed to pick up two very large Pack's cylinders. He picked up the gold on his plugger, annealed it, then placed it in the cavity, and gave it five blows with the automatic mallet. Throughout the whole operation the same plan was followed. Once he made a mistake and used seven blows. A dozen times he used but four blows. When the operation was completed he had a beautiful looking filling, so far as appearances went, but he had so little density in the mass, that the microbes played hide and seek between the different layers of gold which had been placed in the cavity. But he had a beautiful looking operation.

Because the operator just spoken of displayed so much ignorance in condensing his gold is no reason for condemning the use of the automatic mallet. Not in the least, for every once in a while my attention is called to operations which have stood the test of time—say twenty odd years—where the gold was condensed by using the automatic or some other sort of mechanical mallet. And occasionally some of my patients who are in the neighborhood of sixty years of age, will point with pride to operations which were made forty years ago, and which have every appearance of being made but yesterday. They will tell me that no mallet force of any nature was used in condensing the gold. This is very true, but the men who made the operations, unbeknown to themselves, spread the dentine in condensing the gold. The men spoken of had greatest power in their fingers, and thus many of them could do this.

If in condensing gold foil into those cavities on which we operate, the dentine is sprung so that it grasps the gold, all is accomplished which is necessary. The means used for doing this are not of such great importance. What is important, however, is that it be done.

In this short and hastily written essay, altogether too much has been left unsaid which should be said. You have been given some reasons why I endorse the use of the hand mallet. If my conclusions are wrong, if the results of the experiments are incorrect, call my attention to your proofs and they will receive every attention from me. Remember that the more experimental work a person does, the more one fact is impressed on his mind, and it is this, that errors are likely to take place in the most carefully made work. Before I sit down, I wish to illustrate this statement with a little story. I wished the specfic gravity of an experimental filling. My own weighings gave a specific gravity of 17.4. I sent the filling to a well known chemist and asked that he make a specific gravity. You may imagine my horror when forty-eight hours later, he returned the filling with his figures showing a specific gravity of 34.50, and he declared that his figures were correct. I could tell you of several other episodes of a similar nature, but what's the use.—*Dental Review.*

